# Characterization of Platelet-Derived Growth Factor-A Expression in Mouse Tissues Using a lacZ Knock-In Approach

**DOI:** 10.1371/journal.pone.0105477

**Published:** 2014-08-28

**Authors:** Johanna Andrae, Leonor Gouveia, Liqun He, Christer Betsholtz

**Affiliations:** 1 Department of Immunology, Genetics and Pathology, Rudbeck Laboratory, Uppsala University, Uppsala, Sweden; 2 Department of Medical Biochemistry and Biophysics, Division of Vascular Biology, Karolinska Institute, Stockholm, Sweden; Childrens Hospital Los Angeles, United States of America

## Abstract

Expression of the platelet-derived growth factor A-chain gene (*Pdgfa*) occurs widely in the developing mouse, where it is mainly localized to various epithelial and neuronal structures. Until now, *in situ* mRNA hybridization (ISH) has been the only reliable method to identify *Pdgfa* expression in tissue sections or whole mount preparations. Validated protocols for *in situ* detection of PDGF-A protein by immunohistochemistry is lacking. In particular, this has hampered understanding of *Pdgfa* expression pattern in adult tissues, where ISH is technically challenging. Here, we report a gene targeted mouse *Pdgfa* allele, *Pdgfa^ex4^* ^COIN^, which is a combined conditional knockout and reporter allele. Cre-mediated inversion of the COIN cassette inactivates *Pdgfa* coding while simultaneously activating a beta-galactosidase (lacZ) reporter under endogenous *Pdgfa* transcription control. The generated *Pdgfa^ex4^* ^COIN-INV-lacZ^ allele can next be used to identify cells carrying a *Pdgfa* null allele, as well as to map endogenous *Pdgfa* expression. We evaluated the *Pdgfa^ex4^* ^COIN-INV-lacZ^ allele as a reporter for endogenous *Pdgfa* expression patterns in mouse embryos and adults. We conclude that the expression pattern of *Pdgfa^ex4^* ^COIN-INV-lacZ^ recapitulates known expression patterns of *Pdgfa*. We also report on novel embryonic and adult *Pdgfa* expression patterns in the mouse and discuss their implications for *Pdgfa* physiology.

## Introduction

The platelet-derived growth factor (PDGF) family plays fundamental roles during several stages of vertebrate development (reviewed in [1]. The mammalian PDGFs encompass 5 protein isoforms, which are dimers of 4 distinct, but related, polypeptide chains (PDGF-A-D) encoded by separate genes. PDGF-A-D chains assemble into 4 homodimers (PDGF-AA, BB, CC, DD) and one heterodimer (PDGF-AB). PDGFs exert their biological activities through two receptor tyrosine kinases, PDGF receptor-alpha (PDGF-Rα) and beta (PDGF-Rβ) (reviewed in [2]. Whereas ligand-receptor interactions mapped *in vitro* suggest a significant degree of redundancy in PDGF ligand-receptor interaction, *in vivo* gene knockout analyses show that PDGF-AA and PDGF-CC are the principal ligands for PDGF-Rα, at least during development, whereas PDGF-BB is the key ligand for PDGF-Rβ [3]–[7]. The developmental roles of PDGFs mapped to-date suggest paracrine modes of signaling, i.e. PDGFs released from one type of cells act on neighbors of a different type (reviewed in [8]. Thus, various developing epithelia express PDGF-A and PDGF-C, whereas the neighboring mesenchyme expresses PDGF-Rα [9]–[11]. Similarly, PDGF-B is expressed in angiogenic vascular endothelial cells, and triggers responses in adjacent murals cells (vascular smooth muscle cells (VSMC) and pericytes) [12], [13]. The paracrine mode of action of PDGF-AA and PDGF-BB depends in part on their extracellular distribution. This, in turn, is regulated by C-terminal heparan sulfate proteoglycan-binding motifs that may be present or absent in the PDGF protein depending on alterative splicing (in PDGF-A) [14] or alternative proteolytic processing (in PDGF-B) [15]. The activity of PDGF-C and PDGF-D in tissues further depends on extracellular proteolytic processing; both factors carry N-terminal CUB domains that require removal for receptor-binding to occur (reviewed in [16]).

Numerous developmental roles have been elucidated for PDGFs, mainly through studies of knockout mice, but also by using neutralizing antibodies and kinase inhibitors in non-mammalian vertebrates, such as frog [17]–[19] and zebrafish [20]. Developmental roles for PDGFs have also been suggested through studies in sea urchins [21], [22]. The developmental functions of PDGFs indicated through these different studies span from gastrulation (PDGF-A/PDGF-Rα) [17]–[19] to the formation of cranial and cardiac neural crest (PDGF-Rα) [6], [23], epithelial-mesenchymal interactions in organogenesis (PDGF-A/PDGF-Rα) [10], [11], [24], [25], glia development in the central nervous system (PDGF-A/PDGF-Rα) [26], development of the axial skeleton, palate and teeth (PDGF-A/PDGF-C/PDGF-Rα) [6], [7], [27]–[30], the recruitment of vascular mural cells during angiogenesis (PDGF-B/PDGF-Rβ) [12], [13] and hematopoiesis (PDGF-B/PDGF-Rβ) [31]. Thus, PDGFs play numerous developmental roles at different anatomical locations and in different morphogenetic processes. The PDGF and PDGF receptor expression patterns have been assessed in some, but not all, of these processes.

PDGFs have also been implicated in the pathogenesis of a number of different diseases. With few exceptions, mainly involving various cancers, the evidence for involvement is based on correlations between expression and disease. Functional evidence through specific gene inactivation, or the use of highly specific inhibitors, is generally lacking. Nevertheless, a wealth of data suggests the involvement of different PDGFs in different types of fibrotic conditions affecting the lung, liver, skin, kidney and heart (reviewed in [32]). PDGF signaling has also been implicated as a pathogenic driver in vascular disorders, including atherosclerosis, pulmonary hypertension and retinopathy (reviewed in [1]). In all of these conditions, the assumed mode of signaling is paracrine. A similar mechanism has also been proposed for the involvement of PDGFs in the formation of tumor stroma (reviewed in [33]). However, in addition to the paracrine functions, autocrine PDGF signaling is also known to play a role in some cancers. This evidence is particularly strong in the case of dermatofibrosarcoma protuberans, a human skin tumor caused by chromosomal translocations that fuse *PDGFB* coding sequences with transcriptional control elements from the *COL1A1* gene [34]. This leads to production of PDGF-B in collagen-I producing cells (fibroblasts/fibrocytes). These cells carry endogenous PDGF receptors, hence forming the basis for an autocrine growth stimulatory loop.

In determining the mode of action and function of PDGFs in adult tissues in physiological and pathophysiological settings, two hurdles appear: (i) the lack of well-validated tools and techniques for the determination of gene and protein expression (especially the expression of PDGF-A and PDGF-B) and (ii) the lack of specific and validated inhibitors for studies *in vivo*. For PDGF-A, the most broadly expressed of the PDGF ligands, this void is noteworthy: no validated specific immunohistochemistry protocols for *in vivo* PDGF-AA detection have yet been reported, to our knowledge. Moreover, the use of a floxed *Pdgfa* allele has not been reported previously. The embryonic-to-early-postnatal lethality of full *Pdgfa* knockout [5] prevents analysis of adult roles of PDGF-A using this model.

Several PDGF-A antibodies are available commercially, and their use in immunohistochemistry (IHC) has been reported in tissues from human [35]–[37], rat [38], chicken [39], and mouse [40]. To our knowledge, none of the reported PDGF-A immunohistochemistry protocols have been validated using *Pdgfa* knockout tissue as negative control. In theory, even with the access to specific antibodies and staining protocols, the characterization of PDGF-A expression in tissues by IHC is likely going to be problematic since PDGF-A is rapidly secreted from the producer cell. Moreover, most PDGF-A is expressed as a short, diffusible, splice isoform, whereas the long heparan sulfate proteoglycan-binding isoform is rare in most instances [41], [42]. Therefore, developmental expression studies have primarily utilized RNA *in situ* hybridization (ISH). In this way, PDGF-A expression has been mapped to e.g. CNS neurons [43], developing embryonic organs [9], embryonic lung [5], [44] and intestinal epithelium [11], tubular epithelium of testis and epididymis [25], embryonic epidermis and hair follicle epithelium [10]. Whereas in some instances the spatial resolution of non-radioactive ISH has permitted mapping of the expression with single cell resolution, this is usually not the case. Also, ISH techniques are prone to non-specific background signals; in our own hands this was especially problematic in tissues rich in extracellular matrix, as occurs commonly in both normal and pathological adult tissues. Although we successfully applied non-radioactive ISH to uncover embryonic *Pdgfa* mRNA expression patterns in several instances [10], [11], [24], we experienced notorious difficulties in maintaining comparable signal intensities and signal-to-noise ratios from one experiment to the other.

To overcome the mentioned problems in elucidating specific PDGF-A expression patterns and functions, we have now generated, and performed an initial characterization of, a mouse *Pdgfa* allele (*Pdgfa* ^ex4COIN^), which combines the features of a conditional null and expression reporter allele. After Cre-mediated recombination and functional inactivation, the allele (*Pdgfa^ex4^* ^COIN-INV-lacZ^) expresses lacZ from endogenous regulatory elements, thus providing a reliable proxy for *Pdgfa* expression. *Pdgfa^ex4^* ^COIN-INV-lacZ^ also provides a marker for cells in which *Pdgfa* gene inactivation has occurred. This is of great importance since Cre-mediated recombination in somatic cells is generally chimeric. *Pdgfa* ^ex4COIN^ therefore provides a new useful tool for studies of PDGF-A functions in mice, particularly in adults. Here, we show that *Pdgfa* ^ex4COIN^ functions as a conditional null allele. We also use the Cre-recombined allele (*Pdgfa^ex4^* ^COIN-INV-lacZ^) to confirm previously reported embryonic *Pdgfa* expression patterns, as well as to provide new information about *Pdgfa* expression patterns in healthy adults.

## Materials and Methods

### Ethics statement

The *Pdgfa^ex4COIN^* mice were generated at Regeneron Pharmaceuticals Inc**©**, USA, and shipped to Karolinska Institute and Uppsala University, Sweden, were all analyzes were done. The protocol for this study was approved by the Stockholm’s North Committee on the Ethics of Animal Experiments (permit numbers N33/10 and N15/12) and by the Uppsala Committee (permit number C224/12). All efforts were made to minimize animal suffering, and all surgery was performed under Hypnorm/Midazolam anesthesia.

### Generation of mice

The *Pdgfa^ex4COIN^* allele was generated by inserting a TMLacZ-COIN-f1neo cassette as an artificial intron into *Pdgfa* exon 4 in a BAC clone. Exon 4 was thereby split into exon4a (78 bp) and exon4b (110 bp). The lacZ gene was inserted antisense and flanked by lox71 and lox66 sites. These modified loxP sites enable irreversible inversion of the intermediate sequence in the presence of Cre. The engineered BAC was recombined into ES cells with 129S6SvEv/C57BL6F1 background using VelociGene technology (Valenzuela et al., 2002). Two ES cell clones with a correctly integrated *Pdgfa^ex4COIN^* allele (clone B3 and D5) were obtained and used to generate mouse lines that were subsequently confirmed to be indistinguishable. One of these lines (D5) was kept for further analysis.

Mice were genotyped by PCR using the following primer pairs; *Pdgfa^WT^* allele: 5′-TCAGCCCTGTACATTCAAGG-3′ and 5′-GAGCTTCGGGCTAATAACCT-3′ (484 bp); *Pdgfa* ^ex4COIN^ allele: 5′-TCAGCCCTGTACATTCAAGG-3′ and 5′-TTCCCATTCTAAACAACACCCT-3′ (366 bp); *Pdgfa^ex4^* ^COIN-INV-lacZ^ allele: 5′-TCAGCCCTGTACATTCAAGG-3′ and 5′-CACTTGGCACCAGAATGTAG-3′ (680 bp), *EIIa-cre* allele: 5′-GCGGTCTGGCAGTAAAAACTATC-3′ and 5′-GTGAAACAGCATTGCTGTCACTT-3′. Heterozygous *Pdgfa^ex4COIN^* and *Pdgfa^ex4^* ^COIN-INV-lacZ^ mice were bred with C57BL6/J wildtype mice. For statistical analysis of born homozygotes generation F2-F4 mice were used.

### RNA isolation and quantitative real-time PCR (qPCR)

RNeasy Mini Kit (Qiagen) was used to extract mRNA from different mouse tissues (cerebellum, cerebrum, fat, lung, esophagus, liver, stomach, pancreas, spleen, jejunum, colon, kidney, bladder), followed by cDNA synthesis using SuperScript III First-Strand Synthesis SuperMix (Invitrogen), Oligo-dT_20_ and 1 µg of extracted total RNA. qPCR was performed using 100 ng of cDNA and the following Taqman probes: *Pdgfa* (Mm00435540_m1, Applied Biosystems), *lacZ* (customized by Applied Biosystems: primers 5′-GGGAGGTGCCTCTTGATGTG-3′ and 5′-CTGTGGAAGCTCAGGAAGTTCAAT-3′, probe: 5′-CAGCTGCTTGCCTTGTG-3′) and *Elastin* (Mm00514670_m1, Applied Biosystems). Non-template and non-reverse transcriptase controls were included and the reactions were performed using CFX-96 Real Time system (Bio-Rad). Expression results were normalized to the expression of 18 s rRNA endogenous control (X03205.1, Applied Biosystems) and relative quantification was performed using Livak method (2^−ΔΔCt^) [45].

### X-gal staining

Visualization of βeta-galactosidase expression was done in whole mount embryos or dissected organs. X-gal staining of muscle tissue to be further used for IHC was performed on free-floating sections. Embryos were immersion fixed in 4% paraformaldehyde for 1 h. For staining of inner organs, embryos were decapitated and the skin was partially removed before fixation. Postnatal mice (older than P12) were perfusion fixed through the heart for 3 min; inner organs were then cut out and postfixed for 1 h. Prior to X-gal staining, tissues were washed in PBS and permeabilized in PBS/2 mM MgCl_2_/0.02% Igepal/0.01% Na-deoxycholate for at least 1 h at room temperature, with change of the solution 3 times. Staining was performed for 2–16 h at 37°C in 50 mg/ml X-gal (Promega) in PBS/2 mM MgCl_2_/0.02% Igepal/0.01% Na-deoxycholate 5 mM K_4_Fe(CN)_6_/5 mM K_3_Fe(CN)^6^. Washings in PBS ended the reaction.

### Expression analyzes of adult tissues

Tissues from 4-months-old *Pdgfa^ex4COIN/+^* mice and wildtype controls were dissected out, fixed and X-gal stained as above. The following organs were collected; brain, spinal cord, pituitary, eyes, thymus, thyroid, trachea, esophagus, heart, lung, aorta, diaphragm, liver, pancreas, mesentery, stomach, duodenum, jejunum, colon, spleen, kidney, adrenal gland, skin, uterus, ovary, breast gland, testis, epididymis. Tissues were whole-mount X-gal stained, embedded in paraffin, sectioned and weakly counterstained with Mayer’s hematoxylin/eosin. Photos were taken in an AxioImager M2 microscope (Zeiss).

### Immunofluorescence staining of paraffin sections

Whole mount X-gal stained brown adipose tissue and lung tissue from P5 mice was embedded in paraffin, and sectioned in 8 µm thick sections. Sections were deparaffinized through graded series of EtOH, and submitted to heat-induced antigen retrieval in 0.01M-citrate buffer or in Target Retrieval Solution, citrate pH 6.0 (Dako Cytomation, S2369). Blocking was in PBS/1% bovine serum albumin/0.5% triton X-100 for 30–60 min at room temperature. Incubation with primary antibodies was in PBS/0.5% BSA/0.25% triton X-100 at +4°C over night: rabbit-anti-beta-galactosidase (1∶50) (MP Biochemicals/Capell 55976); goat anti-SPC (1∶50) (Santa Cruz, sc7706). Mouse-anti-human ASMA (alpha-smooth muscle actin) conjugated to Cy3 (1∶100) (Sigma C6198) was incubated together with the secondary antibodies. Sections were washed in PBS and incubated with secondary antibodies in PBS/0.5% BSA/0.25% triton X-100 for 30–60 min at room temperature: goat-anti-rabbit Alexa Fluor-488 (1∶200) (Life Technologies); donkey-anti-goat Alexa Fluor-488 (1∶200) (Molecular Probes); donkey-anti-rabbit Alexa Fluor-568 (1∶200) (Molecular Probes). Washed sections were mounted with ProLong Gold anti-fade reagent (Invitrogen). Imaging was done in an SP8 confocal microscope (Leica). X-gal staining was visualized with transmitted light.

### Fluorescent detection of neuromuscular junctions

Whole mount X-gal stained muscle was soaked in 30% sucrose, frozen with dry ice and sectioned in a freezing sleigh microtome. Free-floating 100 µm thick sections were re-stained with X-gal for 30 min to enable the staining to uniformly penetrate the section. Sections were blocked in PBS/1% bovine serum albumin/0.5% triton X-100 for 1 h at room temperature. Sections were stained at room temperature with αlpha-bungarotoxin conjugated to Alexa Fluor-555 (Molecular Probes) diluted to 3 ng/µl in PBS/0.5% BSA/0.25% triton X-100 followed by washings in PBS and mounting in ProLong Gold anti-fade reagent with DAPI (Invitrogen). Imaging was done using a LSM700 confocal microscope (Zeiss). Muscle morphology and positive X-gal staining were visualized using transmitted light in the empty A647 channel.

### *Pdgfa* gene expression in public databases

Expressed Sequence Tag (EST) data were extracted from the EST profile of the NCBI UniGene databases (http://www.ncbi.nlm.nih.gov/UniGene/). Human *PDGFA* expression data was extracted from Hs.535898, and mouse *Pdgfa* data from Mm.2675.

### Statistics

The genotype distribution of mice that were born alive, from heterozygous crossings of *Pdgfa^ex4COIN-INV-lacZ/+^* was compared to the expected Mendelian distribution. Pups from >10 litters were compared using Chi-square test (www.graphpad.com). P<0.05 was considered to be statistically significant.

## Results

### Generation of a conditional *Pdgfa^ex4^* ^COIN^ allele

**Figure 1:**
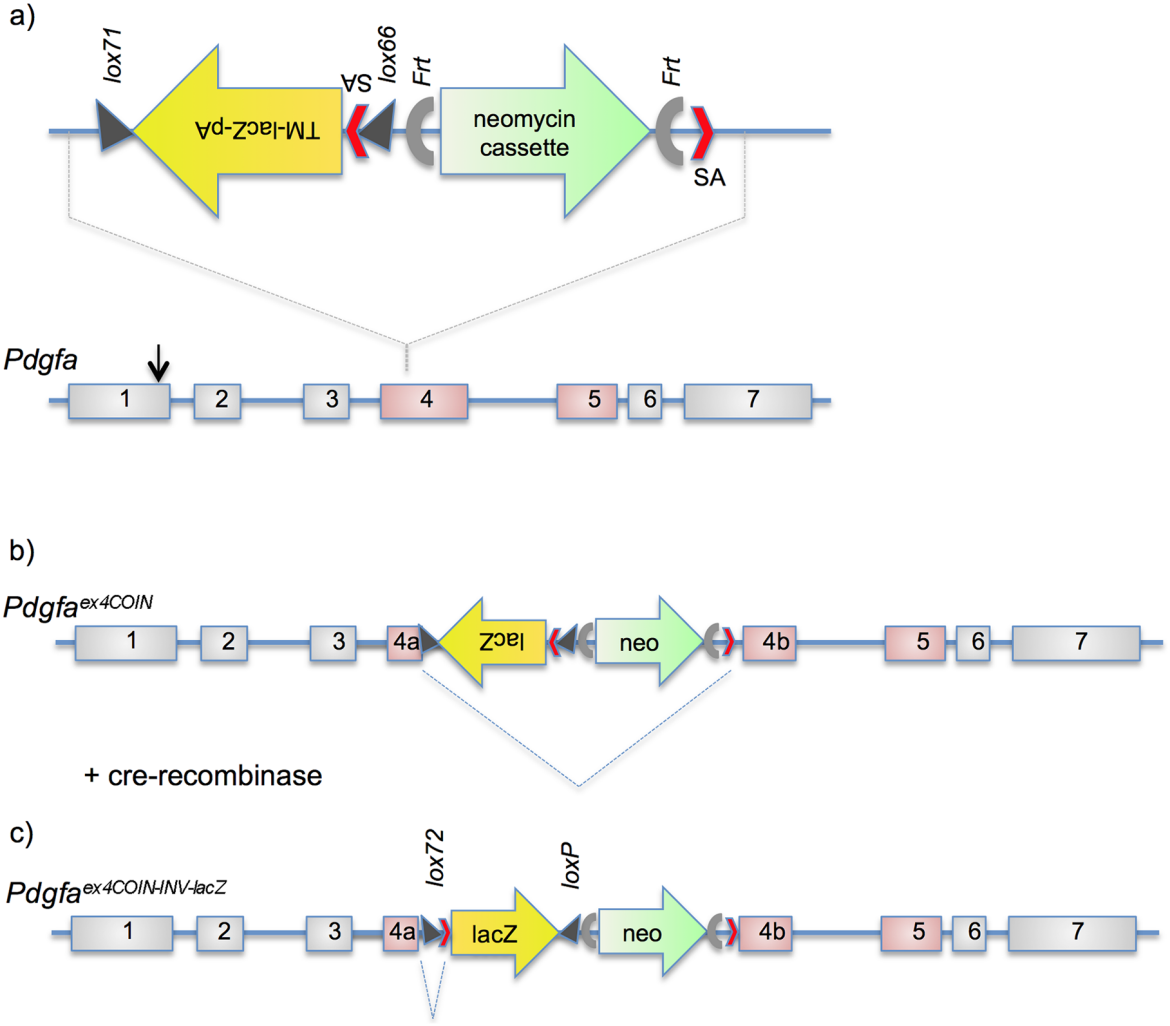
Strategy for the construction of *Pdgfa^ex4COIN^* and *Pdgfa^ex4COIN-INV-lacZ^* alleles. (a) Outline of the COIN module introduced as an artificial intron in the middle of *Pdgfa* exon 4. Abbreviations: TM, transmembrane; pA, polyA; SA, splice acceptor. (b) *Pdgfa^ex4COIN^* allele with the lacZ cassette in anti-sense orientation. The expected splicing that rejoins exon 4a and exon 4b in the *Pdgfa^ex4COIN^* transcript is indicated. (c) The *Pdgfa^ex4COIN-INV-lacZ^* allele following Cre-mediated inversion of the lacZ cassette. Splicing from exon 4a now enters into the lacZ cassette and transcription terminates at its polyA site.

A conditional *Pdgfa* knockout allele was generated using the “conditional-by-inversion” (COIN) strategy [46], [47]. This strategy is based on the insertion of an inverted (and inactive) COIN module into the gene of interest, by a single targeting event in embryonic stem (ES) cells. In *Pdgfa^ex4^* ^COIN^, the COIN module, along with a neomycin cassette, was placed as an artificial intron into *Pdgfa* exon 4. As a result, exon 4 was split in two new exons, 4a and 4b (Fig. 1). The *Pdgfa^ex4^* ^COIN^ allele was expected to be functional, since splicing between exons 4a and 4b resulted in an RNA sequence identical to that encoded by the original exon 4. Exon 4-encoded sequences are absolutely required for the production of a functional PDGF-A protein, and thus, this splicing event is necessary for the functionality of the *Pdgfa^ex4^* ^COIN^ allele. Two targeted *Pdgfa^ex4^* ^COIN^ carrying lines were generated using VelociGene technology [48], and both lines were bred to homozygosity (*Pdgfa^ex4^* ^COIN/ex4COIN^). Initial experiments confirmed that the two lines behaved identically, and one was therefore selected for further breeding. *Pdgfa^ex4^* ^COIN/ex4COIN^ mice were born in Mendelian numbers (data not shown) and found to be viable and normal (followed up to 10 months) as expected for a functional *Pdgfa* allele [5].

### Generation of the *Pdgfa^ex4^* ^COIN-INV-lacZ^ allele

**Figure 2:**
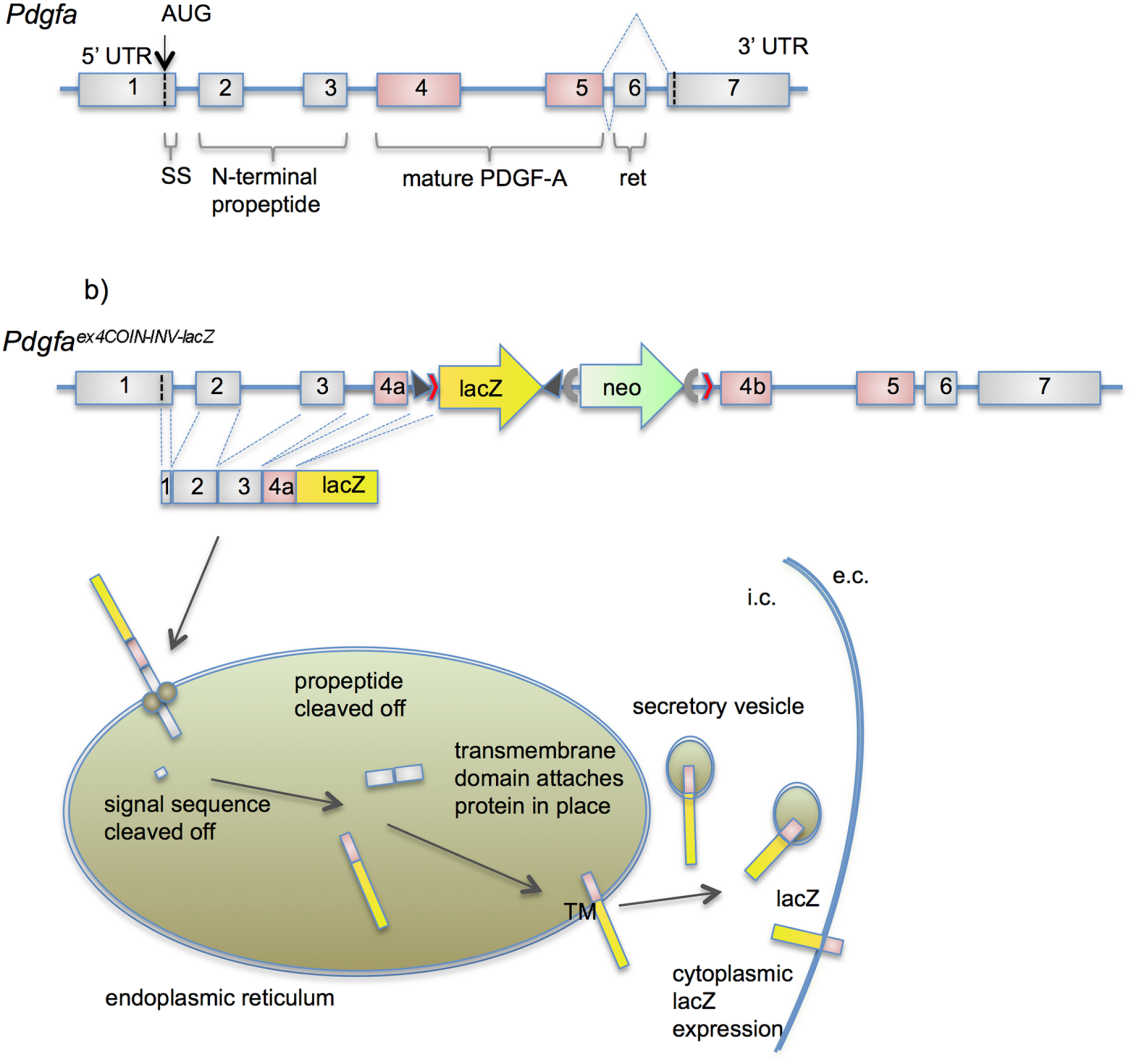
Processing of a PDGF-A-lacZ fusion reporter protein in *Pdgfa^ex4COIN-INV-lacZ^* expressing cells. (a) Schematic outline of the *Pdgfa* exons and their contribution to the normal PDGF-A protein precursor. (b) Predicted transcription, translation and processing of the PDGF-A-lacZ fusion protein. The normal PDGF-A pre-pro and pro-peptide processing sites remain in the fusion protein. After exon1-encoded signal sequence removal, the exons 2-3-encoded pro-peptide is cleaved. The lacZ transmembrane domain anchors the exon 4a-lacZ fusion protein to the plasma membrane with the lacZ catalytic domain facing the cytoplasm. i.c, intracellular; e.c extracellular; UTR, untranslated region; SS, signal sequence; ret, extracellular matrix-binding retention motif.

The COIN-module was flanked by *lox66* and *lox71* sites oriented head-to-head (Fig. 1). Therefore, Cre recombinase was expected to mediate irreversible inversion of the COIN-module, resulting in fusion of the lacZ sequences with *Pdgfa* coding sequences (Fig. 1). We crossed *Pdgfa^ex4^* ^COIN^ mice with the Cre deleter strain EIIa-Cre (Xu et al., 2001) and identified offspring with an inverted COIN-module using PCR. The resulting *Pdgfa^ex4^* ^COIN-INV-lacZ^ allele was expected to be a null allele. Exon 4a was predicted to splice into the activated COIN module and, as a result, produce a fusion protein consisting of an N-terminal exon 4a-derived portion of PDGF-A, a transmembrane (TM) domain, a lacZ cassette, and a polyadenylation site. A schematic illustration of the expected expression and processing of the PDGF-Aex4a-TM-lacZ fusion protein is shown in Figure 2. As expected from a *Pdgfa* null allele, no homozygous *Pdgfa^ex4^* ^COIN-INV-lacZ/*ex4*COIN-INV-lacZ^ mice were found alive after birth (Table 1).

**Table 1:** Genotype distribution from heterozygous crossings of *Pdgfa* ^ex4COIN-INV-lacZ^.

Pdgfa^ex4COIN-INV-lacZ^×Pdgfa^ex4COIN-INV-lacZ^			
+/+	lacZ/+	lacZ/lacZ	Chi-square
16 (34%)	31 (66%)	-	p = 0.0004

Figures indicate number of individuals (percentage in brackets).

**Figure 3:**
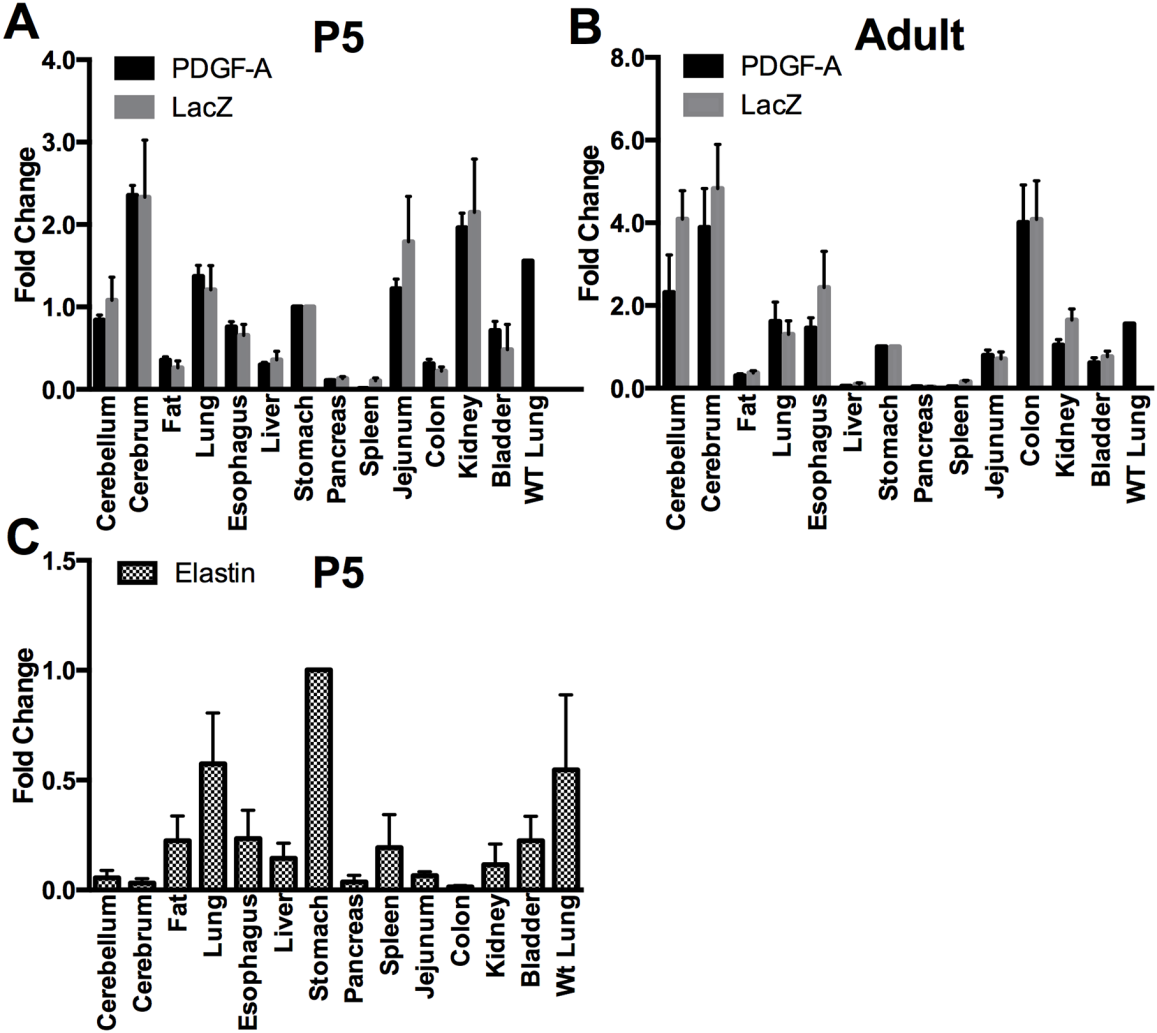
Comparison of *Pdgfa*, *lacZ* and *Elastin* mRNA expression in selected organs of *Pdgfa* ^ex4COIN-INV-lacZ/+^ mice. Quantitative PCR analyzes of *Pdgfa* and *lacZ* in P5 (a) and adult (b) organs. The relative expression of the two genes follows the same pattern in different organs. Stomach was used as the reference sample (mean+SD) and its values set to 1.0. Wild-type lung mRNA was used as a negative control for *lacZ*. (c) Expression of *Elastin* in P5 organs shows a different expression pattern compared to *Pdgfa* and *lacZ*, as expected. Fold change on y-axis, Error bars show the standard deviation.

Since no deletion of endogenous *Pdgfa* genomic sequences occurred in the *Pdgfa^ex4^* ^COIN-INV-lacZ^ allele, we expected the encoded PDGF-Aex4a-TM-lacZ fusion protein to reproduce the endogenous *Pdgfa* expression pattern. In order to confirm that *Pdgfa* and *Pdgfa^ex4^* ^COIN-INV-lacZ^ were similarly expressed, we compared quantitative real-time PCR (qPCR) data for *Pdgfa* and *LacZ* across a panel of tissues. Using Taqman probes against *Pdgfa* and *LacZ* the relative levels of expression of the wildtype *Pdgfa* and the *Pdgfa^ex4^* ^COIN-INV-lacZ^ alleles in different organs were compared in *Pdgfa^ex4^* ^COIN-INV-lacZ/+^ mice at two different ages, P5 and adult. This showed that *Pdgfa* and *lacZ* expression had highly similar organ distribution at both ages (Fig. 3a, b). Lung tissue from a wildtype control showed no *lacZ* expression, as expected (Fig. 3a, b). To further validate the comparison, an *Elastin* Taqman probe was used to asses an irrelevant gene in the the same RNA samples. As expected, *Elastin* mRNA showed a completely different relative organ distribution (Fig. 3c). Furthermore, high levels of *Pdgfa* and *lacZ* mRNA expression correlated well with stronger X-gal staining, e.g. in kidney, lung and brain at P5 (as described later in this paper).

### Developmental expression of *Pdgfa^ex4^* ^COIN-INV-lacZ^

**Figure 4:**
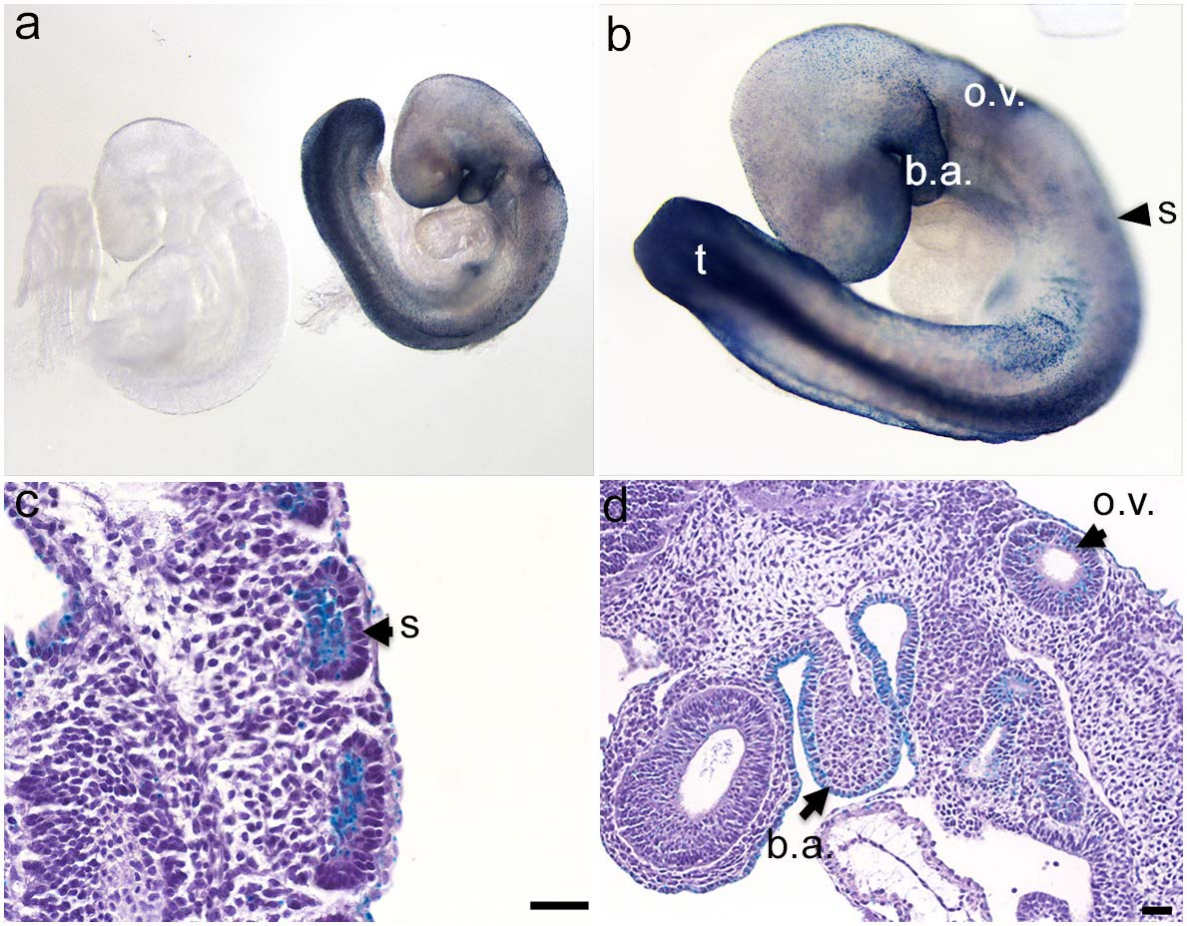
Embryonic expression of *Pdgfa^ex4COIN-INV-lacZ^* at E9.5. (a) X-gal staining of an E9.5 *Pdgfa^ex4COIN-INV-lacZ^* embryo (right) and a wildtype littermate control (left). (b) X-gal staining is concentrated to 1^st^ branchial arch, the otic vesicles, somites and tail. (c, d) Longitudinal sections of paraffin embedded embryo. (c) *Pdgfa^ex4COIN-INV-lacZ^* expression in somites. (d) Expression in epithelia of e.g. otic vesicle and 1^st^ branchial arch. b.a., 1^st^ branchial arch; o.v., otic vesicle; s, somites; t, tail. Scale bar 50 µm.

**Figure 5:**
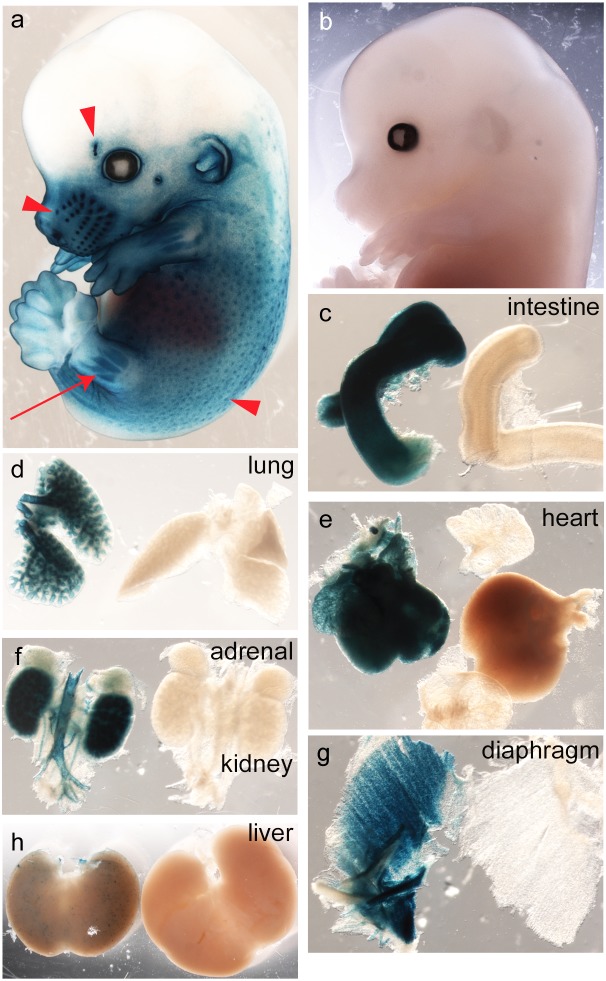
Embryonic expression of *Pdgfa^ex4COIN-INV-lacZ^* in E14.5 whole embryo/organs. Whole mount X-gal staining of an E14.5 (a) *Pdgfa^ex4COIN-INV-lacZ/+^* embryo, arrow points at developing hindlimb muscles, arrowheads points at eye brow, whisker hair follicles, back skin hair follicles. (b) *Pdgfa^+/+^* littermate. Note the absence of any X-gal staining. (c–g) Overnight X-gal staining of individual intact organs dissected from E14.5 *Pdgfa^ex4COIN-INV-lacZ/+^* (with blue staining) and *Pdgfa^+/+^* (no staining) embryos. (c) jejunum, (d) lung, (e) heart (atria partially detached from the *Pdgfa^+/+^* heart) (f) kidneys flanking the aorta and branches, (g) diaphragm, (h) liver.

**Figure 6:**
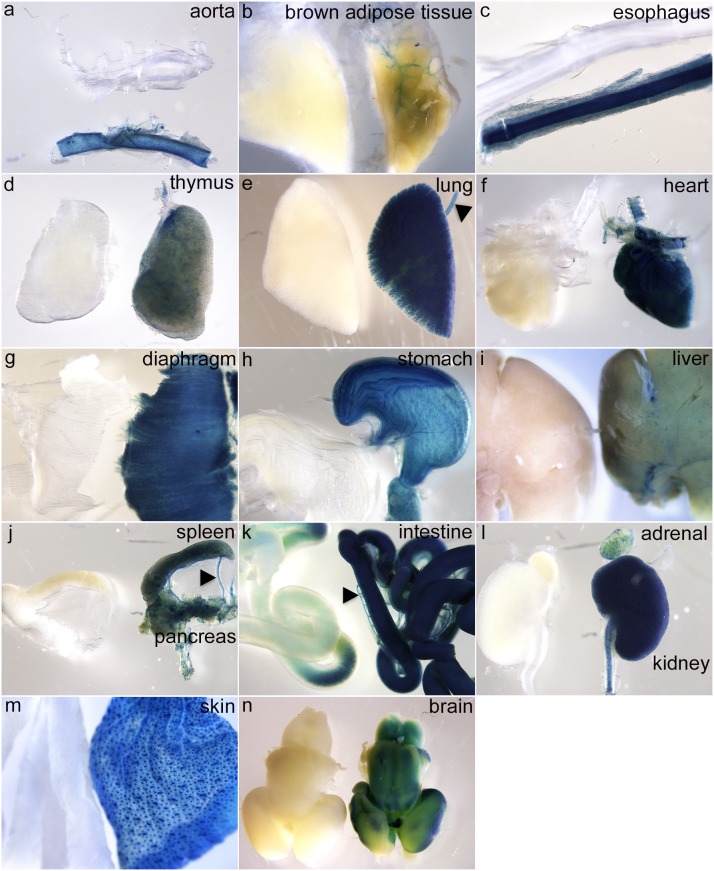
Embryonic expression of *Pdgfa^ex4COIN-INV-lacZ^* in E17.5 whole organs. X-gal staining of whole organs from E17.5 *Pdgfa^ex4COIN-INV-lacZ/+^* embryos (to the right or below) and wildtype littermate controls (to the left or on top). (a) aorta, (b) brown adipose tissue – note expression in associated vasculature, (c) esophagus, (d) thymus, (e) lung and trachea (arrowhead), (f) heart, (g) diaphragm, (h) stomach, (i) liver, (j), spleen, pancreas and mesenteric vessels (arrow head), (k) intestine, mesenteric vessels (arrow head), (l) adrenal gland and kidney, (m) skin, (n) brain.

**Figure 7:**
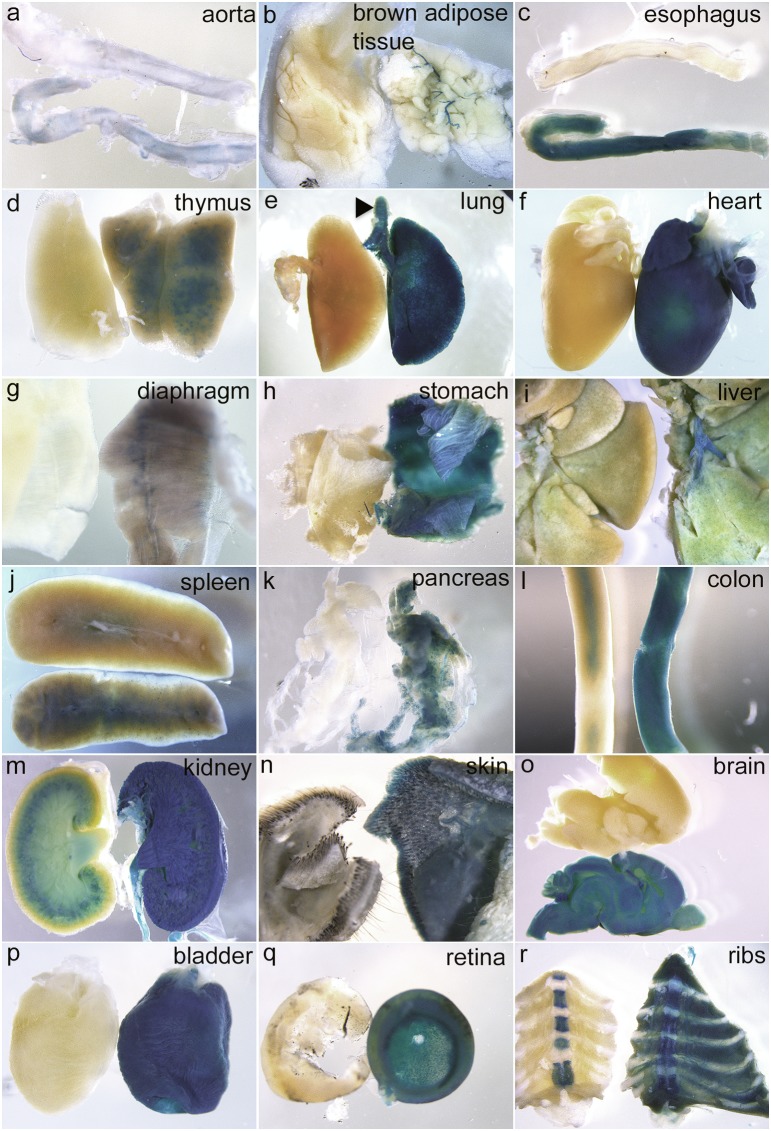
Postnatal expression of *Pdgfa^ex4COIN-INV-lacZ^* in P5 whole organs. X-gal staining of whole organs from P5 *Pdgfa^ex4COIN-INV-lacZ/+^* pups (to the right or below) and wildtype littermate controls (left or on top). (a) aorta, (b) brown adipose tissue – note expression in associated vasculature, (c) esophagus, (d) thymus, (e) lung, trachea (arrow head), (f) heart, (g) diaphragm, (h) stomach, (i) liver, (j) spleen, (k) pancreas, (l) colon, (m) kidney, (n) skin, (o) brain, (p) urinary bladder, (q) retina, (r) ribs.

Since the *Pdgfa* expression pattern has previously been mapped at relatively high detail during mouse embryogenesis, we first analyzed heterozygous *Pdgfa^ex4^* ^COIN-INV-lacZ/+^ mouse embryos for *lacZ* expression by X-gal staining. Analysis of embryonic day (E) 9.5, E14.5 and E17.5 *Pdgfa^ex4^* ^COIN-INV-lacZ/+^ mice confirmed many of the *Pdgfa* mRNA expression patterns that have previously been demonstrated by ISH (summarized in Table 2). Whole mount X-gal staining of E9.5 *Pdgfa^ex4^* ^COIN-INV-lacZ/+^ embryos showed region specific expression in e.g. the 1^st^ branchial arch, the otic vesicles, in somites and in the tail (Fig. 4). Whole-mount X-gal staining of E14.5 *Pdgfa^ex4^* ^COIN-INV-lacZ/+^ embryos showed distinct expression in developing hair follicles in the back skin, in whiskers and eyebrows (Fig. 5a, arrowheads). Distinct staining was also observed in developing skeletal muscle, e.g. in limbs and in the thoracic region (Fig. 5a red arrow). *Pdgfa^+/+^* littermate controls were completely negative for X-gal staining at this age (Fig. 5b). To enable proper penetration of the X-gal staining solution, inner organs were dissected out and individually stained over night. Strong staining was confirmed in developing intestine (Fig. 5c), lung (Fig. 5d), heart (Fig. 5e) kidneys (Fig. 5f) and skeletal muscle (Fig. 5g). At the whole-mount level, strong X-gal staining was also observed in large arteries (e.g. the aorta, Fig. 5f). Apposed tissues with strong and negative/weak staining were observed at the whole-mount level. For example, positive lung epithelium neighbored negative mesenchyme (Fig. 5d), and strongly positive kidney tissue neighbored the weakly positive adrenal gland (Fig. 5f). The liver displayed a weak punctuate staining, which was deemed specific since no staining was observed in *Pdgfa^+/+^* liver (Fig. 5h). Whole organ X-gal stainings of freely dissected organs were repeated at E17.5 and P5 with consistent results (Fig. 6 and 7). *Pdgfa^ex4^* ^COIN-INV-lacZ^ expression was detected in aorta, vessels, esophagus, thymus, lung, heart, diaphragm, stomach, liver, spleen, pancreas, intestine, adrenal, kidney, skin, bladder and brain. At P5, *Pdgfa^ex4^* ^COIN-INV-lacZ^ expression was also detected in the retina (Fig. 7q). Background expression due to endogenous beta-galactosidase activity was detected in the intestine (Fig. 6k and 7l), kidney (Fig. 7m) and in the sternum (Fig. 7r). In summary, the X-gal staining pattern in heterozygous *Pdgfa^ex4^* ^COIN-INV-lacZ/+^ embryos appeared to reproduce known expression patterns of endogenous *Pdgfa* previously mapped using non-radioactive ISH (Table 2). This fact, combined with the strength and localized nature of the staining, suggests that the *Pdgfa^ex4^* ^COIN-INV-lacZ^ expression reports endogenous *Pdgfa* expression faithfully.

**Table 2:** Summary of previously published data on *Pdgfa* expression compared with expression patterns revealed by X-gal staining of *Pdgfa^ex4COIN-INV-lacZ^* mice.

Pdgfa^ex4COIN-INV-lacZ^ expression pattern	Previous method	Species (Age)	Reference
CNS neurons e.g. Purkinje cells	ISH (^35^S)	mouse (E18.5, adult)	[43]
Exocrine pancreas			
Dorsal root ganglia			
	IHC	mouse (E11.5)	[56]
Developing eye, lung, hair, somites,muscle	ISH (^35^S)	mouse (E7.5-E14.5)	[9]
Otic vesicle			
Medullary papilla of kidney	ISH (^35^S), IHC	human (fetal, adult)	[57]
Bronchial epithelium of lung	ISH (a.p.)	mouse (E14.5)	[5]
Corneal and eyelid epithelium	ISH (^35^S)	mouse (E16, P7)	[58]
Scattered cells in the liver	ISH (^35^S)	human	[59]
Hair follicles	ISH (a.p.)	mouse (E15.5–E17.5)	[10]
Neuromuscular junctions	IHC	mouse, human (adult)	[54]
Testis, epididymis	ISH (a.p.)	mouse (E17.5, P30)	[25]
Intestinal epithelium of jejunum	ISH (a.p.)	mouse (E15.5, P30)	[11]
Gastric epithelium of the stomach			
Somites, myotome	ISH (a.p.)	mouse (E10.5)	[27]
Cardiac muscle	IHC	chicken, quail (fetal)	[60]

a.p. - alkaline phosphatase.

**Figure 8:**
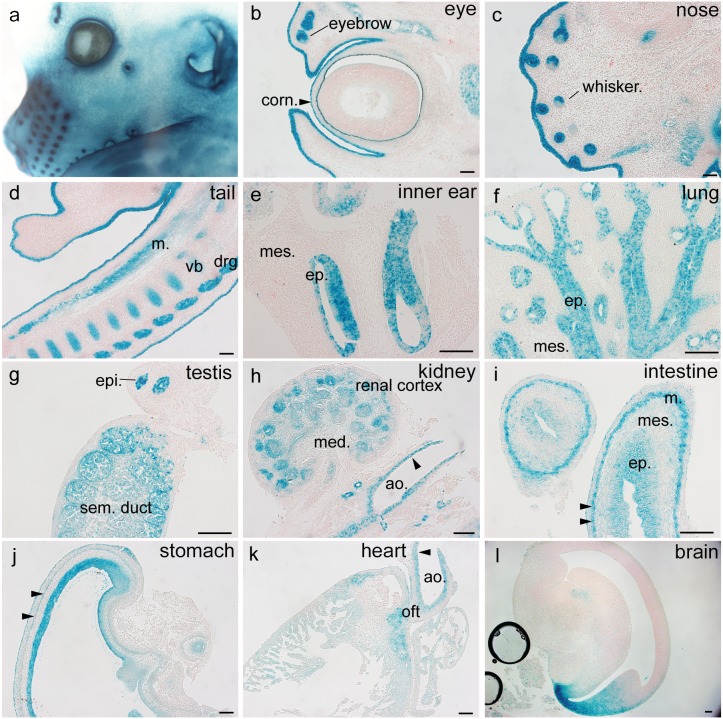
Embryonic expression of *Pdgfa^ex4COIN-INV-lacZ^* in E14.5 tissue sections. Paraffin sections of developing organs at E14.5, counterstained with nuclear fast red. (a) Close-up of the head region from the whole mount stained *Pdgfa^ex4COIN-INV-lacZ/+^* embryo in Fig. 3a. Note expression in hair follicles and in surface ectoderm. Strong staining occurs in the ectoderm covering developing eyelids and outer ear. (b–l) Sections from different regions/organs of a *Pdgfa^ex4COIN-INV-lacZ/+^* embryo. (b) Eye and surrounding tissue; expression is seen in cornea (arrowhead), eyelid ectoderm, eyebrow follicles and lacrimal gland. (c) Expression in whisker hair follicles, epidermis, and developing facial muscles. (d) Expression in the tail and genital tubercle ectoderm (m, skeletal muscle; vb, cartilage primordium of vertebral body; drg, dorsal root ganglia). (e) Expression in the inner ear (ep, epithelium of otic vesicles; mes, mesenchyme). (f) Expression in bronchial epithelium of lung. (g) Expression in testis seminipherous ducts and mesonephric duct in epididymis (epi.). (h) Expression in developing renal epithelium and surrounding structures (med, medulla; ao. aorta). (i) Developing jejunum. Expression in pseudostratified epithelium (ep.) and developing muscular layers (m, arrow heads; mes., mesenchyme). (j) In the stomach, expression is mainly seen in the epithelium and developing muscle layers (arrowheads). (k) Expression in the heart, particularly in the outflow tract (oft). Arrowheads in k and h point at aortic VSMC. (l) Sagittal section of the developing brain showing expression in the dorsal horn of the lateral ventricle, and in amygdaloid and hippocampal epithelium. Scale bars 50 µm.

In order to map the *Pdgfa^ex4^* ^COIN-INV-lacZ^ expression pattern at higher resolution, we analyzed sections from whole-mount stained E14.5 organs (Fig. 8). Previous work has established that *Pdgfa* mRNA is expressed broadly in embryonic epithelia, in skeletal, cardiac and smooth muscle, and in neuronal cells (Table 2) (reviewed in [1]. These expression patterns were confirmed and extended. We found *Pdgfa^ex4^* ^COIN-INV-lacZ^ expression in dermal keratinocytes and hair follicle epithelial cells (Fig. 8a, b, c, d), corneal epithelium (Fig. 8b arrowhead), developing inner ear epithelium (Fig. 8e), bronchial epithelium (Fig. 8f), testis and epididymis epithelium (Fig. 8g), renal epithelium (Fig. 8h), and intestinal and stomach epithelium (Fig. 8i, j). We further documented *Pdgfa^ex4^* ^COIN-INV-lacZ^ expression in developing visceral smooth muscle (Fig. 8i, j arrowheads), skeletal muscle (Fig. 8d), and cardiac muscle, the latter particularly strong in the cardiac outflow tract (Fig. 8k), and in vascular smooth muscle (Fig. 8h, k arrow heads). In the E14.5 brain, *Pdgfa^ex4^* ^COIN-INV-lacZ^ expression was restricted to neuroepithelial tissue around the dorsal horn of the lateral ventricle (Fig. 8l).

### Adult expression of *Pdgfa^ex4^* ^COIN-INV-lacZ^

While the observed patterns of *lacZ* expression in *Pdgfa^ex4^* ^COIN-INV-lacZ^ embryos were consistent with, and confirmatory of, already published patterns of *Pdgfa* mRNA expression in the mouse embryo, only limited information is available about *Pdgfa* expression patterns in adult mammals. The occurrence of *PDGFA/Pdgfa* sequences in public human and mouse EST databases suggests widespread but weak expression in many organs (Table 3), but this information does not reveal the cellular source of expression. Moreover, it is not clear if the lack of sequences in certain organs/tissues reflects a true lack of expression, or that expression in underrepresented cells has gone undetected due to dilution.

**Table 3:** *PDGFA/Pdgfa* expression based on publicly available expressed sequence tag (EST) data.

	Human		Mouse	
Tissue	*PDGFA*sequences	total librarysize	*Pdgfa*sequences	total librarysize
brain	2	1092688	5	475384
connective tissue	2	149072	0	19807
embryonic tissue	2	212896	25	677554
esophagus	1	20154	NA	NA
Extraembryonictissue	NA	NA	2	74703
eye	4	208840	3	185387
heart	2	89524	1	54558
intestine	2	231981	6	86859
kidney	1	210778	3	123578
liver	1	205291	0	111370
lung	1	334815	3	99799
mammary gland	1	151230	8	303048
mouth	1	66150	NA	NA
nerve	2	15535	NA	NA
ovary	3	101488	1	54858
pancreas	3	213440	8	106229
pituitary gland	0	16526	1	18069
placenta	1	283019	NA	NA
prostate	1	189536	1	29507
skin	0	210759	2	118925
spleen	0	53397	3	92417
testis	2	435204	3	121820
thymus	0	79697	1	121153
thyroid	0	46583	2	8820
uterus	1	232093	2	6855

Data were extracted from the NCBI UniGene database. The “Tissue” column indicates which organs/tissues from which the EST libraries were made in alphabetical order. The “*PDGFA/Pdgfa* sequences” columns show the number of the *PDGFA/Pdgfa* EST sequences found in the organs/tissues in human and mouse, respectively. The “total library size” column shows the total number of EST sequences from the organs/tissues. “NA” values in the table indicate that corresponding organ/tissue data were not available in that species. Organs/tissues lacking *PDGFA/Pdgfa* EST sequences in both human and mouse are not shown.

**Figure 9:**
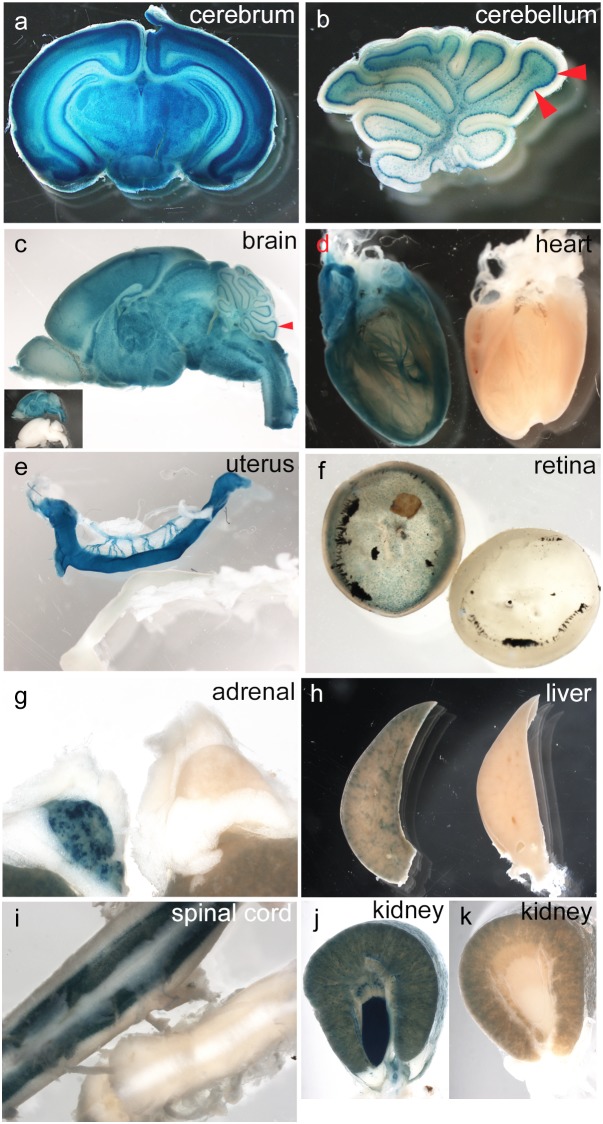
Expression of *Pdgfa^ex4COIN-INV-lacZ^* in adult whole organs. Whole mount X-gal staining of postnatal whole organs or organ slices. Organs were cut or sliced before staining. (c–i) Tissues from *Pdgfa^ex4COIN-INV-lacZ/+^* and *Pdgfa^+/+^* littermates. *Pdgfa^+/+^* negative controls appear in (c–i) and (k) to the right or below of the *Pdgfa^ex4COIN-INV-lacZ/+^* sample. (a) Coronal slice of a P12 cerebrum shows specific staining in neuronal layers. (b) Sagittal section of a P12 cerebellum. Arrowheads indicate Purkinje cell layer. (c) Sagittal section of a P15 brain. Inserted picture shows the same brain together with negative control. (d) P15 heart. (e) P15 uterus. Note staining of uterine blood vessel VSMC. (f) P60 retina. (g) P60 adrenal glands. (h) Slice of P60 liver lobe. (i) Dorsal view of P60 spinal cord. (j) P60 *Pdgfa^ex4COIN-INV-lacZ/+^* kidney slice, note the strong staining in the medullary papilla. (k) P60 *Pdgfa^+/+^* kidney slice, note background staining in the cortex.

X-gal staining of adult brain slices (Fig. 9a–c) showed a complex, mainly neuronal, pattern of *Pdgfa^ex4^* ^COIN-INV-lacZ^ expression, with clear variation between different neuronal subgroups. For example, cerebellar Purkinje cells were one example of neurons with strong *Pdgfa^ex4^* ^COIN-INV-lacZ^ expression (Fig. 9b, c arrow heads). At a gross level, the adult cerebral and cerebellar *Pdgfa^ex4^* ^COIN-INV-lacZ^ expression pattern confirms the pattern of *Pdgfa* mRNA expression previously reported in the adult mouse brain using radioactive ISH [43]. In several other organs, such as the heart (Fig. 9d) and uterus (Fig. 9e), X-gal expression was relatively uniform at the level of a whole-mount perspective, consistent with the low cell type complexity in these organs compared to the brain. In yet other adult organs, *Pdgfa^ex4^* ^COIN-INV-lacZ^ expression was obviously non-uniform, and displayed distinctive cell type or region specificities, e.g. in the retina (Fig. 9f), adrenal gland (Fig. 9g), liver (Fig. 9h), spinal cord (Fig. 9i) and kidney (Fig. 9j; note the strong staining in the medullary papilla). Whereas the corresponding wildtype tissues were for the most part negative for X-gal staining, some endogenous background was noticed in a few tissues, including cartilage (not shown), kidney cortex (Fig. 9k) and intestinal lumen (not shown). The latter likely represents intestinal bacterial staining; it was seen only in association with intestinal lumen content.

### Cell-type specific expression of *Pdgfa^ex4^* ^COIN-INV-lacZ^ in adult tissues

**Figure 10:**
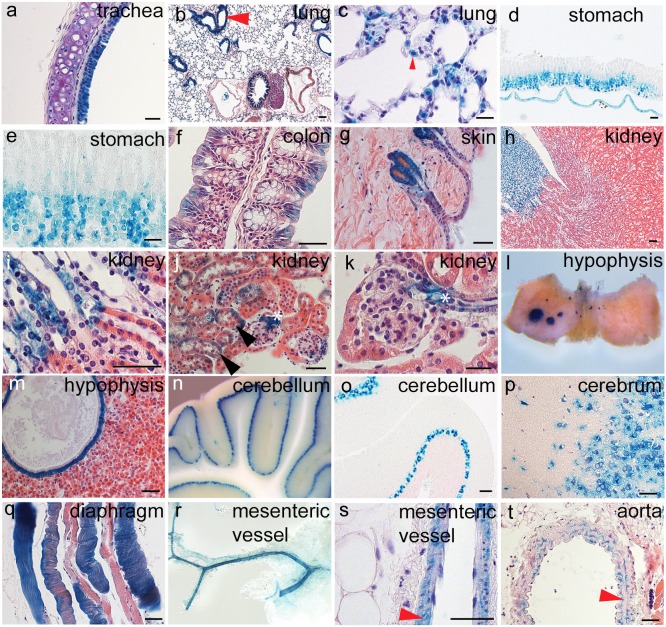
Cell type-specific expression of *Pdgfa^ex4COIN-INV-lacZ^* in adult organs. Expression of *Pdgfa^ex4COIN-INV-lacZ^* in organs and organ sections from a 4-month-old male. (a) Expression in hyaline cartilage and respiratory epithelium around the trachea. (b) Expression in lung, arrowhead points at bronchi, note the strong epithelial staining. (c) Lung alveolar region, arrowhead indicate positive cell with the morphological appearance of a type II pneumocyte. (d) Stomach. Note staining in the mucosa and in the underlying muscular layer. (e) Stomach epithelium, staining at the base of the gastric pits. (f) Colon. Note staining in surface enterocytes. (g) Hair follicle epithelium. (h) Kidney medulla with strong expression in the renal papilla. (i) Expression in Henley’s loop cells in the renal papilla. (j) Expression in tubular epithelium and the juxtaglomerular apparatus in the kidney cortex. (k) Close view of expression in the juxtaglomerular apparatus (white asterisk). (l) Dissected pituitary, whole mount stained, note expression in Rathke’s cleft cysts (RCC). (m) Ciliated epithelium of RCC. (n, o) Cerebellum. Expression restricted to Purkinje cells. (p) Expression in forebrain neurons. (q) Expression in diaphragm muscle fibers. (r) Expression in mesenteric arteries. (s) Expression in mesenteric arterial VSMC. (t) Expression in aortic VSMC. Scalebars 50 µm (except figures b, d, n where scale bars are 25 µm).

In order to provide details about the cellular patterns of *Pdgfa* expression in adult mice, whole mount X-gal stained tissues were sectioned and counterstained with hematoxylin and eosin. Tissues from more than twenty-five different organs were analyzed in this way. This confirmed the general patterns of cell-type specific expression of *Pdgfa^ex4^* ^COIN-INV-lacZ^ observed in embryos, namely in various types of epithelia, muscle, and neuronal tissue (Fig. 10).

Epithelial expression of *Pdgfa^ex4^* ^COIN-INV-lacZ^ was observed throughout the adult body. Expression levels appeared variable and often regionally restricted, implicating localized regulation of expression and possibly also region-specific functions for the produced PDGF-A protein. In the lung, *Pdgfa^ex4^* ^COIN-INV-lacZ^ expression was observed in the respiratory epithelium in the trachea (Fig. 10a), in the epithelium of main bronchi and terminal bronchioles (Fig. 10b, red arrowhead), and in alveoli (Fig. 10c). In the latter, expression was non-uniform and localized mainly to cells resembling type II pneumocytes (Fig. 10c, red arrowhead). Epithelial expression of *Pdgfa^ex4^* ^COIN-INV-lacZ^ was also observed throughout the gastrointestinal tract. Also here, epithelial expression was non-uniform. In the stomach, expression was mainly observed in the corpus, where it sub-localized to cells at the base of the gastric glands (Fig. 10d, e). In the colon, expression was instead localized in the surface epithelial cells, whereas crypts were negative, or showed low expression (Fig. 10f). In the skin, expression was observed in the basal layer of keratinocytes, as well as in hair follicle epithelial cells (Fig. 10g). In the kidney, expression was particularly strong in Henle’s loop epithelium in the renal papilla (Fig. 10h, i), but weaker expression was also observed in cells in the distal tubules in the cortex (Fig. 10j, arrowheads). An unexpected location of *Pdgfa^ex4^* ^COIN-INV-lacZ^ epithelial expression was asymmetrically located cysts in the pituitary (Fig. 10l, m). These cysts were lined with ciliated epithelium, making them reminiscent of Rathke’s cleft cysts (RCC), which are benign remnants of Rathke’s cleft, the embryonic origin of the anterior pituitary lobe. RCC have been described in humans where they are often asymptomatic [49]. RCC in mice have been reported previously [50].

Similar to the epithelial expression, the adult neuronal expression of *Pdgfa^ex4^* ^COIN-INV-lacZ^ was widespread, but non-uniform at the cellular level. In the cerebellum, strong and specific expression was observed in Purkinje neurons, whereas no other neuronal population was positive in this part of the brain (Fig. 10 n, o). The complex and widespread cellular pattern of expression of *Pdgfa^ex4^* ^COIN-INV-lacZ^ in the cerebrum was primarily neuronal (Fig. 10p), but similar to the situation in the cerebellum, not all neuronal populations were positive.

Muscular expression of *Pdgfa^ex4^* ^COIN-INV-lacZ^ was observed in skeletal muscle, as exemplified by muscle cells in the diaphragm in (Fig. 10q), in cardiomyocytes (Fig. 9d and data not shown) and in VSMC, as illustrated in mesenteric arteries (Fig. 10r, s arrowhead), the aorta (Fig. 10t arrowhead) and in bronchial arteries (Fig. 10b). Also in muscle cells, expression was non-uniform, as illustrated e.g. in the kidney, where it was conspicuous in the arteriolar VSMC associated with the juxtaglomerular apparatus (Fig. 10j, k asterisk).

### *Pdgfa^ex4^* ^COIN-INV-lacZ^ expression at neuromuscular junctions

**Figure 11:**
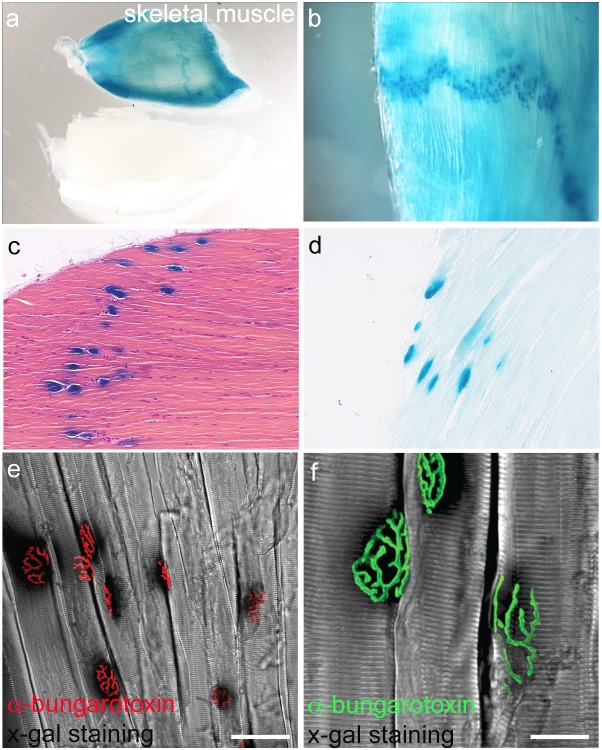
Expression of *Pdgfa^ex4COIN-INV-lacZ^* in neuromuscular junctions. (a) Whole mount X-gal staining of musculus quadriceps femoris from P20 *Pdgfa^ex4COIN-INV-lacZ/+^* and *Pdgfa^+/+^* littermates. (b) Higher magnification shows a band staining coinciding with the location of neuromuscular junctions. (c) Paraffin section of X-gal stained P60 *Pdgfa^ex4COIN-INV-lacZ/+^* muscle, counterstained with hematoxylin-eosin. (d) Paraffin section of X-gal stained P60 muscle, weakly counterstained with nuclear fast red. (e) Confocal imaging of free-floating sections of P60 muscle, stained with X-gal (seen as black shadows) and alpha-bungarotoxin (red). (f) Confocal z-stack of X-gal stained P60 muscle with superimposed alpha-bungarotoxin staining (green). Scale bar 50 µm in (e) and 25 µm in (f).

PDGF-A is produced by cultured myoblasts [51] and developing skeletal muscle [9], [27]. We confirmed the expression of *Pdgfa^ex4^* ^COIN-INV-lacZ^ in both embryonic (Fig. 5g) and adult (Fig. 10o) skeletal muscle. However, similar to the epithelial cells and neurons, X-gal staining was non-uniform. Analysis of femoral quadriceps muscles from *Pdgfa^ex4^* ^COIN-INV-lacZ^ and wildtype controls revealed two distinct expression patterns. First, there was a general and uniform X-gal staining in all muscle fibers, which was not seen in the PDGF-A^+/+^ littermate control (Fig. 11a). Second, we observed a band of intensely stained spots stretching across the approximate middle of the muscle (Fig. 11b, c, d). Similar bands of stained spots were seen also in other muscles, including the diaphragm (Fig. 7g). This staining was clearly visible already after 30 minutes of X-gal incubation, at which time the more general staining was undetectable or weak (Fig. 11c, d). The localization of the spots suggested a correlation with neuromuscular junctions. Indeed, visualization of the neuromuscular junctions using Alexa Fluor-555-conjugated alpha-bungarotoxin, which binds to acetylcholine receptors, provided a spatial correlation with the X-gal staining (Fig. 11e, f). The X-gal staining was localized to the postsynaptic area of the muscle fiber, suggesting the expression of *Pdgfa^ex4^* ^COIN-INV-lacZ^ from local synaptic muscle cell nuclei.

### Localization of *Pdgfa^ex4^* ^COIN-INV-lacZ^ expression to specific cell types

**Figure 12:**
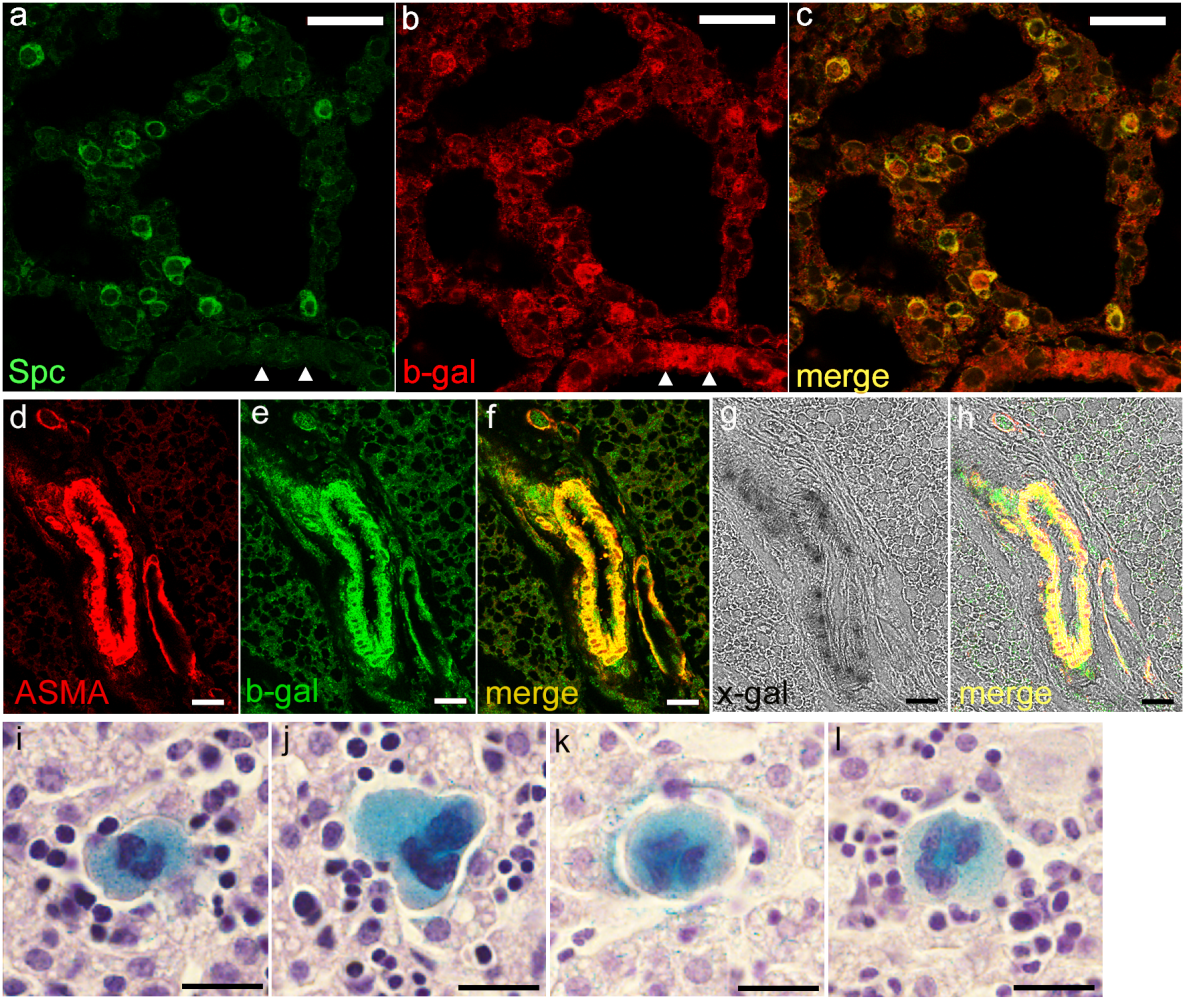
Cell-type specific expression in P5 *Pdgfa^ex4COIN-INV-lacZ^* mice. (a–c) Immunofluorescence labeling of Surfactant protein-C (SPC) and beta-galactosidase in lung. Note co-expression in SPC positive type II pneumocytes, whereas bronchial epithelium is beta-galactosidase positive only (arrowheads in a and b). (d–h) Co-expression of alpha-smooth muscle actin (ASMA) and beta-galactosidase in vascular smooth muscle cells of blood vessels in brown adipose tissue. (d–f) Immunofluorescent stainings of (d) ASMA and (e) beta-galactosidase. (g) X-gal staining visualized with transmitted light overlap with ASMA and b-gal (h). (i–l) X-gal staining (blue) in megakaryocytes in liver, counterstained with hematoxylin/eosin. Scale bar 25 µm.

The expression of *Pdgfa^ex4^* ^COIN-INV-lacZ^ enables localization to individual cells. We used co-immunofluorescence stainings of paraffin embedded tissue from P5 mice, to confirm expression in type-II pneumocytes and in vascular smooth muscle cells (Fig. 12). Surfactant protein-C (SPC) co-localized with beta-galactosidase in individual cells in the alveolar walls of the lung (Fig. 12a–c). Importantly, beta-galactosidase expression was also detected in the bronchial epithelium, where no SPC was expressed (Fig. 12a, b arrowheads). In vessels of brown adipose tissue, alpha-smooth muscle actin was co-expressed with beta-galactosidase (Fig. 12d–f). The fluorescent staining overlapped with the X-gal staining, as shown with transmitted light in the confocal microscope (Fig. 12g, h). Expression of *Pdgfa^ex4^* ^COIN-INV-lacZ^ could also be localized to specific cell-types based on morphology. In the liver, strong X-gal staining was detected in megakaryocytes (Fig. 12i–l) confirming previous data on the expression of PDGF genes during megakaryoblastic differentiation [52].

## Discussion

We report on the generation and first analysis of a conditional null and expression reporter *Pdgfa* allele. The allele was generated using the COIN technique pioneered by scientists at Regeneron Pharmaceuticals [46]. We started by validating that the *Pdgfa^ex4^* ^COIN^ allele was functional by assessing viability and lack of phenotypes associated with PDGF-A deficiency in homozygous *Pdgfa^ex4^* ^COIN/ex4COIN^ mice. We also confirmed that mice homozygous for the Cre-activated allele *Pdgfa^ex4^* ^COIN-INV-lacZ^ were not recovered after birth, as expected for *Pdgfa* null mice on C57Bl6 enriched genetic background. The early postnatal viability originally reported for *Pdgfa* null mice was observed only in mixed C57Bl6/129Ola hybrid background [5].

We next analyzed heterozygous *Pdgfa^ex4^* ^COIN-INV-lacZ/+^ mice as a potential tool for *Pdgfa* expression analysis, utilizing the lacZ reporter gene inserted into the *Pdgfa* locus. No endogenous genomic sequences were deleted in the *Pdgfa^ex4^* ^COIN^ or *Pdgfa^ex4^* ^COIN-INV-lacZ^ alleles, and hence we were hopeful that the expression of the *lacZ*-gene from *Pdgfa^ex4^* ^COIN-INV-lacZ^ would faithfully reproduce the endogenous *Pdgfa* expression pattern. Indeed, using qPCR analysis, we confirmed that the mRNA levels of *Pdgfa* and *lacZ* showed highly similar relative expression levels in different organs, suggesting co-regulation.

PDGF-A is a secreted protein and we therefore aimed for a fusion protein strategy in order to minimize potential deviation from the endogenous pattern of expression. A transmembrane anchoring sequence was inserted, such that the encoded PDGF-A-lacZ fusion protein would become membrane-associated in the expressing cells, with the lacZ domain facing the cytoplasmic compartment. Consequently, X-gal staining would be predicted to mark the cytoplasm of *Pdgfa* expressing cells. Indeed, our analysis of embryos showed that expression of *Pdgfa^ex4^* ^COIN-INV-lacZ^ reproduced the patterns of *Pdgfa* expression that have previously been revealed through ISH analysis. This, together with the strength of the lacZ expression from the *Pdgfa^ex4^* ^COIN-INV-lacZ^ allele, and the ease with which it could be localized to specific cell types and individual cells, imply that *Pdgfa^ex4^* ^COIN-INV-lacZ^ is a faithful and powerful *Pdgfa* expression reporter in the mouse. While our data suggest that X-gal staining of *Pdgfa^ex4^* ^COIN-INV-lacZ/+^ mice provides a sensitive and specific proxy for the expression of *Pdgfa*, the model is less useful for other purposes, such as cell sorting or fate mapping (of Pdgfa-expressing cells). For an overview of the features and advantages with the COIN technique, the reader is referred to the original publication by Economides et al [46].

The possibility to map *Pdgfa* expression patterns in adult tissues is of particular interest, since, until now, validated tools and protocols for *in situ Pdgfa* expression analysis in adult mice have not been available. We found abundant *Pdgfa^ex4^* ^COIN-INV-lacZ^ expression in most analyzed adult organs, which were mapped to distinct cell types and even individual cells. The general tissue/cell type pattern of expression was similar in the adult and embryo, i.e. the predominant sites of expression were various epithelial, muscle, and neuronal cell types. The constitutive expression of *Pdgfa^ex4^* ^COIN-INV-lacZ^ in quiescent adult tissues challenges the view of PDGF-AA as being mainly a mitogen for mesenchymal cells during development and tissue repair or pathology, such as wound healing, fibrosis and cancer. Indeed, available information on the transcriptional regulation of the PDGF-A gene largely depicts transcriptional elements engaged by mitogenic signaling, tissue injury and tumor promotion (reviewed in [53]). The transcriptional mechanisms behind the normal constitutive cell-type specific expression of *Pdgfa* observed in the present study remain unknown. Future *in vivo* analysis of *Pdgfa* transcriptional regulation will therefore benefit from the access to faithful gene expression reporters, such as *Pdgfa^ex4^* ^COIN-INV-lacZ^. Moreover, studies on the role of PDGF-A in cancer, including autocrine growth regulation in the cancer cells themselves, as well as the paracrine recruitment of tumor stroma (reviewed in [33], and the involvement of PDGF-A in tissue fibrosis (reviewed in [32]) will benefit from more precise information about the endogenous PDGF-A expression patterns in both normal and pathological situations.

Two conspicuous physiological expression patterns of *Pdgfa^ex4^* ^COIN-INV-lacZ^ illustrate the power of *Pdgfa^ex4^* ^COIN-INV-lacZ/+^ mice for *Pdgfa* expression analysis. 1) The *Pdgfa* expression in pituitary RCC’s remains functionally unclear but provides a possibility for their easy visualization. This may be of use for the analysis of RCC localization and number in correlation with other developmental abnormalities and pathological processes. The *Pdgfa^ex4^* ^COIN-INV-lacZ^ expression in these structures is probably a remnant of the developmental situation in which *Pdgfa* is broadly expressed in the pharyngeal epithelium. 2) The second remarkable *Pdgfa^ex4^* ^COIN-INV-lacZ^ pattern localized to the neuromuscular junctions. Whereas PDGF-A and PDGF-Rα proteins have been suggested at neuromuscular junctions based on IHC techniques [54], the cellular sources of the proteins were not revealed in this study. The *Pdgfa^ex4^* ^COIN-INV-lacZ^ pattern is suggestive in this regard, since the X-gal staining was localized to a region of the muscle fiber corresponding to the postsynaptic area. This expression pattern appears consistent with that of other molecules localized to the postsynaptic membrane of the neuromuscular junction, such as acetylcholine receptors (reviewed in [55]). These observations therefore suggest that *Pdgfa* expression from local (synaptic) nuclei is induced and maintained by synaptic activity and postsynaptic signaling. Further studies using the conditional nature of the *Pdgfa^ex4^* ^COIN^ allele has the potential to reveal the functional importance of *Pdgfa* expression at this location.
