# Correction: Characterization of Platelet-Derived Growth Factor-A Expression in Mouse Tissues Using a lacZ Knock-In Approach

**DOI:** 10.1371/journal.pone.0113204

**Published:** 2014-11-10

**Authors:** The PLOS ONE Staff

The following information is missing from the Funding section: JA received funding from Magnus Bergvall Foundation (http://www.magnbergvallsstiftelse.nu). The funder had no role in study design, data collection and analysis, decision to publish, or preparation of the manuscript.
